# Combinations of common SNPs of the transporter gene ABCB1 influence apparent bioavailability, but not renal elimination of oral digoxin

**DOI:** 10.1038/s41598-020-69326-y

**Published:** 2020-07-27

**Authors:** Chih-hsuan Hsin, Marc S. Stoffel, Malaz Gazzaz, Elke Schaeffeler, Matthias Schwab, Uwe Fuhr, Max Taubert

**Affiliations:** 1 Faculty of Medicine and University Hospital Cologne, Center for Pharmacology, Department I of Pharmacology, University of Cologne, Cologne, Germany; 2 Dr. Margarete-Fischer-Bosch Institute of Clinical Pharmacology, Stuttgart, Germany; 3 University of Tuebingen, Tuebingen, Germany; 4 Department of Clinical Pharmacy, College of Pharmacy, Umm Al-Qura University, Makkah, Saudi Arabia; 5 Department of Clinical Pharmacology, University of Tuebingen, Tuebingen, Germany; 6 Department of Pharmacy and Biochemistry, University of Tuebingen, Tuebingen, Germany

**Keywords:** Clinical pharmacology, Pharmacogenetics, Pharmacokinetics, Haplotypes, Medical research

## Abstract

Effects of different genotypes on the pharmacokinetics of probe substrates may support their use as phenotyping agents for the activity of the respective enzyme or transporter. Digoxin is recommended as a probe substrate to assess the activity of the transporter P-glycoprotein (P-gp) in humans. Current studies on the individual effects of three commonly investigated single nucleotide polymorphisms (SNPs) of the *ABCB1* gene encoding P-gp (C1236T, G2677T/A, and C3435T) on digoxin pharmacokinetics are inconclusive. Since SNPs are in incomplete linkage disequilibrium, considering combinations of these SNPs might be necessary to assess the role of polymorphisms in digoxin pharmacokinetics accurately. In this study, the relationship between SNP combinations and digoxin pharmacokinetics was explored via a population pharmacokinetic approach in 40 volunteers who received oral doses of 0.5 mg digoxin. Concerning the SNPs 1236/2677/3435, the following combinations were evaluated: CGC, CGT, and TTT. Carriers of CGC/CGT and TTT/TTT had 35% higher apparent bioavailability compared to the reference group CGC/CGC, while no difference was seen in CGC/TTT carriers. No significant effect on renal clearance was observed. The population pharmacokinetic model supports the use of oral digoxin as a phenotyping substrate of intestinal P-gp, but not to assess renal P-gp activity.

## Introduction

During the last decades, membrane transporters demonstrated to play an essential role in the pharmacokinetics (PK) and -dynamics (PD) of many drugs, potentially explaining drug-drug interactions and pharmacogenetic sources of variability in drug effects^1–3^.

P-glycoprotein (P-gp), encoded by the gene *ABCB1*, is the first well-characterized membrane transporter^4^. It belongs to the ATP-Binding Cassette (ABC) family and acts as an efflux transporter located in the canalicular side of hepatocytes, the apical membrane of intestinal cells, the luminal side of the tubular cells in the kidney, as well as the apical membrane of brain microvessel endothelial cells, and placental syncytiotrophoblasts^5,6^. Effects of P-gp activity on plasma concentrations of a broad spectrum of endogenous and xenobiotic substances have been shown, including testosterone, aldosterone, antiviral drugs, anticancer drugs, immunosuppressant agents, antifungals and cardiovascular drugs^7–13^. Moreover, many in vivo studies showed that the induction or inhibition of P-gp could cause clinically relevant drug-drug interactions^14^.

To investigate transporter-based drug-drug interactions (TDDIs) of new compounds, digoxin is recommended as a P-gp phenotyping drug in humans by regulatory agencies^15,16^. Digoxin is a cardiac glycoside drug used in congestive heart failure and atrial fibrillation. The bioavailability of oral digoxin is approximately 60–80%, and digoxin is mainly eliminated via glomerular filtration and active tubular secretion, with only minor contribution of non-cytochrome P450 enzymes and fecal excretion^17,18^. In 1999, Greiner et al. demonstrated the importance of intestinal P-gp in its net transport of digoxin across the gut wall by administering digoxin orally and intravenously in combination with rifampin, a strong P-gp inducer^19^. Furthermore, by showing that the co-administration of the P-gp inhibitor verapamil orally and digoxin intravenously resulted in a significant decrease in renal clearance, Pedersen et al. provided first evidence that renal P-gp might play an important role in digoxin elimination^20^. However, recent evidence indicates that renal organic anion transporter polypeptide 4C1 (OATP4C1) might be rate-limiting for renal elimination of digoxin^21–23^, although inhibition of OATP4C1 might be too weak to fully explain the reported effect of verapamil on digoxin clearance^24^. Besides, Sato et al. showed that ritonavir strongly inhibits OATP4C1 in vitro with an IC_50_ of 8.5 µM, indicating a potential for clinically relevant drug-drug interactions. However, a recent clinical trial reported by Penzak et al. showed that the renal clearance of digoxin did not significantly change when administering digoxin orally before and after 14 days of oral ritonavir administration^25^. Overall, it is not clear to which extent P-gp and OATP4C1 determine the renal elimination of digoxin.

Beyond TDDIs, *ABCB1* polymorphisms might have an impact on P-gp activity and respective phenotyping results. Particularly, three commonly investigated single nucleotide polymorphisms (SNPs) in the protein-coding region, i.e., C1236T (rs1128503), G2677T/A (rs2032582) and C3435T (rs1045642), have been associated with changes in P-gp activity in vivo^26–43^ (See Supplementary Table S1). Reported SNP frequencies of C1236T, G2677T/A and C3435T in Caucasians are 0.459, 0.464/0.067 and 0.561, respectively^26^. Of these three SNPs, C1236T and C3435T are silent/synonymous mutations. In the following, combinations of the SNPs are described by three letters indicating the respective nucleic acids at positions 1236, 2677, and 3435, respectively. When referring to individual loci, these are identified by the cDNA position followed by the respective nucleic acid. For genotype identification, two letters are used for the two alleles. In vitro, C1236T does not affect protein expression or mRNA stability^44^. However, mRNA and protein expression levels of *ABCB1/*P-gp for SNP C3435T were decreased in the duodenum and kidney^44,45^ and these silent SNPs might also affect P-gp function by altering protein folding with a reduction of ATP binding affinity or ATP hydrolysis^46^.

In contrast, SNP G2677T/A is a nonsynonymous mutation with substitution of the amino acid alanine with serine or threonine^8^, which was related to a higher transporter activity in NIH-3T3 GP + E86 cells transfected with P-gp cDNA containing G2677T with 47% lower digoxin accumulation compared to G2677G despite a similar protein expression of P-gp^47^. In contrast, Morita et al. did not find statistically significant differences in transcellular transport ratios and intracellular concentrations of digoxin in LLC-PK1 cells transfected with P-gp cDNA for G2677G, G2677T, and G2677A^48^.

The haplotype structure of *ABCB1* SNPs has been analyzed for Caucasian, African-American, Asian-American, and Mexican-American populations^49^. Leschiziner et al. analyzed the haplotype for linkage disequilibrium (LD) of SNPs in *ABCB1* with a minor allele frequency (MAF) ≥ 0.5 and a genotyping success rate ≥ 75% in 47 Caucasians by estimating D′ and the correlation coefficient (*r*^2^) between G2677T/C3435T and other SNPs in *ABCB1*. The D′ value is an absolute standardized linkage disequilibrium coefficient representing the correlation between alleles of a haplotype, conditional on allele frequencies. C3435T/G2677T and C3435T/C1236T had a D′ value of 0.89 and 0.82, respectively, and the D′ value for G2677/C1236T was 0.94, indicating a clear association. Furthermore, C3435T/G2677T and C3435T/C1236T were correlated (*r*^2^ = 0.51 and 0.37, respectively). In addition, G2677T was highly correlated with C1236T (r^2^ = 0.76)^50^. Thus, any of the SNPs C1236T, G2677T and C3435T might be used as a reasonable albeit not fully reliable tagging SNP for the evaluation of a genotype effect.

Hoffmeyer et al. provided the first evidence on pharmacogenetic influences of P-gp activity in humans. Particularly, compared to wild-type, 3435TT carriers of P-gp were shown to have a 38% increase in peak steady-state concentrations (C_max_) after administration of 0.25 mg digoxin (*p* = 0.006) in Caucasians^30^, suggesting a reduced intestinal secretion of digoxin and a reduced activity of P-gp related to the variant. However, in another early study, Gerloff et al. reported that the initial area under the curve (AUC_0–4 h_) and C_max_ values for 1 mg orally administrated digoxin in Caucasians who carried 2677TT and 3435TT were not significantly higher than in subjects with 2677GG and 3435CC^27^.

Until today, numerous evaluations on the genotype/haplotype effect of *ABCB1* on the pharmacokinetic parameters of digoxin in vivo have been published, and the results from 17 previous studies are summarized in Supplementary Table S1. The included publications were identified using PubMed by the search term “(ABCB1 OR MDR1 OR pgp) AND (variant OR polymorphism) AND pharmacokinetics AND digoxin”. In total, 37 articles were identified. Of these, seven were review articles, five had no PK data evaluation, four were not related to digoxin, three were in vitro results, one was not in English, and in two cases the original articles could not be retrieved. Two additional relevant publications from the reference list of the review articles were additionally included.

Overall, the effects of the three *ABCB1* SNPs, C1236T, G2677T/A, and C3435T, on digoxin pharmacokinetics are inconclusive^2^, which might also be a consequence of considering only one of the 3 SNPs to define genotypes. Since the SNPs are in incomplete linkage disequilibrium, considering the SNP combination might be necessary to assess the role of common polymorphisms in digoxin pharmacokinetics accurately^47,49^.

The SNP combinations CGC and TTT at positions 1236/2677/3435, respectively, are the most frequent combinations in the Caucasian population^49^. In contrast to the assumption that the TTT combination might be related to a lower P-gp activity, Dickens et al. demonstrated that triple SNP variants (C1236T, G2677T, C3435T) expressed in *Xenopus laevis* oocytes had no effect on the efflux of digoxin. The efflux rate of digoxin after 45 min was 77.6 ± 11.9% in the triple SNP variant and 82.7 ± 13.6% for wild-type *ABCB1*^51^.

To provide additional information on the effect of common *ABCB1* SNP combinations on P-gp activity in vivo, we investigated the relationship between digoxin pharmacokinetics and SNP combinations in healthy Caucasian subjects using population pharmacokinetic modeling.

## Results

### Summary of subject demographics

Data from 40 healthy Caucasian subjects (22 female) with a mean age of 38 years (range 20–68), a mean height of 1.73 m (range 1.54–1.92 m), and mean body weight of 73.1 kg (range 54.4–97.0 kg) were available for the population pharmacokinetic analysis. All subjects were assigned to four SNP combination groups based on the genotype of P-gp. The SNP combinations were CGC/CGC, CGC/CGT, CGC/TTT, and TTT/TTT (as the respective combination of 1236/2677/3435 SNPs) with 9, 5, 15 and 9 subjects, respectively (see Table 1). Two subjects had different SNP combinations, CGT/TTT and CGT/CTT, and were excluded from the genotype covariate analysis due to the small group size. SNP frequencies of C1236T (0.425), G2677T (0.438) and C3435T (0.525) in the study population were similar to published data^26^, and did not show a significant deviation from Hardy–Weinberg equilibrium (${\chi }^{2}$ of 1.32, 0.745 and < 0.001, respectively). For the SNP pairs C3435T/G2677T, C3435T/C1236T and G2677T/C1236T, a pronounced linkage disequilibrium was found (D′ of 0.78, 1.00, and 1.00; *r*^2^ of 0.55, 0.67 and 0.95, respectively).

**Table 1:** Observed distribution of genotypes for ABCB1.

Abbreviated description of SNP combination group	Number of subjects		Combinations of variants
	Trial I	Trial II	
CGC/CGC^a^	3	6	1236 C/C + 2677 G/G + 3435 C/C
CGC/CGT^a,b^	2	3	1236 C/C + 2677 G/G + 3435 C/T
CGC/TTT^a^	8	7	1236 C/T + 2677 G/T + 3435 C/T
TTT/TTT^a^	3	6	1236 T/T + 2677 T/T + 3435 T/T
–		1	1236 C/T + 2677 G/T + 3435 T/T
–		1	1236 C/C + 2677 G/T + 3435 T/T

^a^38 out of forty subjects in the two trials were included in our evaluation of the effect of SNP combinations. P-gp, P-glycoprotein.

^b^Contains one non-compliant subject.

### Digoxin empirical model

As a base model, a two compartment model with mixed first- and zero-order absorption and linear elimination described the data best. A summary of key model development steps is shown in Table 2. Visual predictive checks (VPCs) indicated a difference in apparent bioavailability and absorption shape between the two trials that was not captured well by the base model. Consequently, a significantly lower bioavailability was identified in trial I compared to trial II (-30.3%, drop in objective function value (OFV) by 72.6) and a significantly lower first-order absorption rate constant (Ka) (0.201 h^−1^ vs. 0.636 h^−1^, drop in OFV by 46.0) was identified in the test period of trial II compared to trial I and the reference period of trial II. When introducing additional estimates for bioavailability in trial I and for Ka in the test period of trial II, VPCs did not show any further misspecification.

**Table 2:** Model selection: Summary of covariate building steps for digoxin pharmacokinetics.

Model	Description	OFV	AIC
**Base model**			
1	2-Compartment model with mixed-order absorption and linear elimination	− 1,166.40	− 1,072.41
**Full model-selection of demographic and physiological covariates**			
2	Base model with separate estimates of bioavailability for different trials	− 1,238.98	− 1,142.98
3	Model 2 with additional separate estimates for first-order absorption rate constants of test period of trial II	− 1,284.96	− 1,183.58
**Final mode with covariate**			
4	Final model with separate estimates of bioavailability according to CGC/CGC, CGC/CGT, CGC/TTT and TTT/TTT ABCB1 SNP combination groups	− 1,311.09	− 1,206.58

Summary of population pharmacokinetic model selection. Starting from the base model with a 2-compartment model, separate estimates for bioavailability and first-order absorption rate constants were introduced into the model. Finally, covariates on the bioavailability of different SNP combination were computed. OFV, objective function value; AIC, Akaike information criterion.

### Covariate model for effects of *ABCB1* SNP combinations

**Figure 1:**
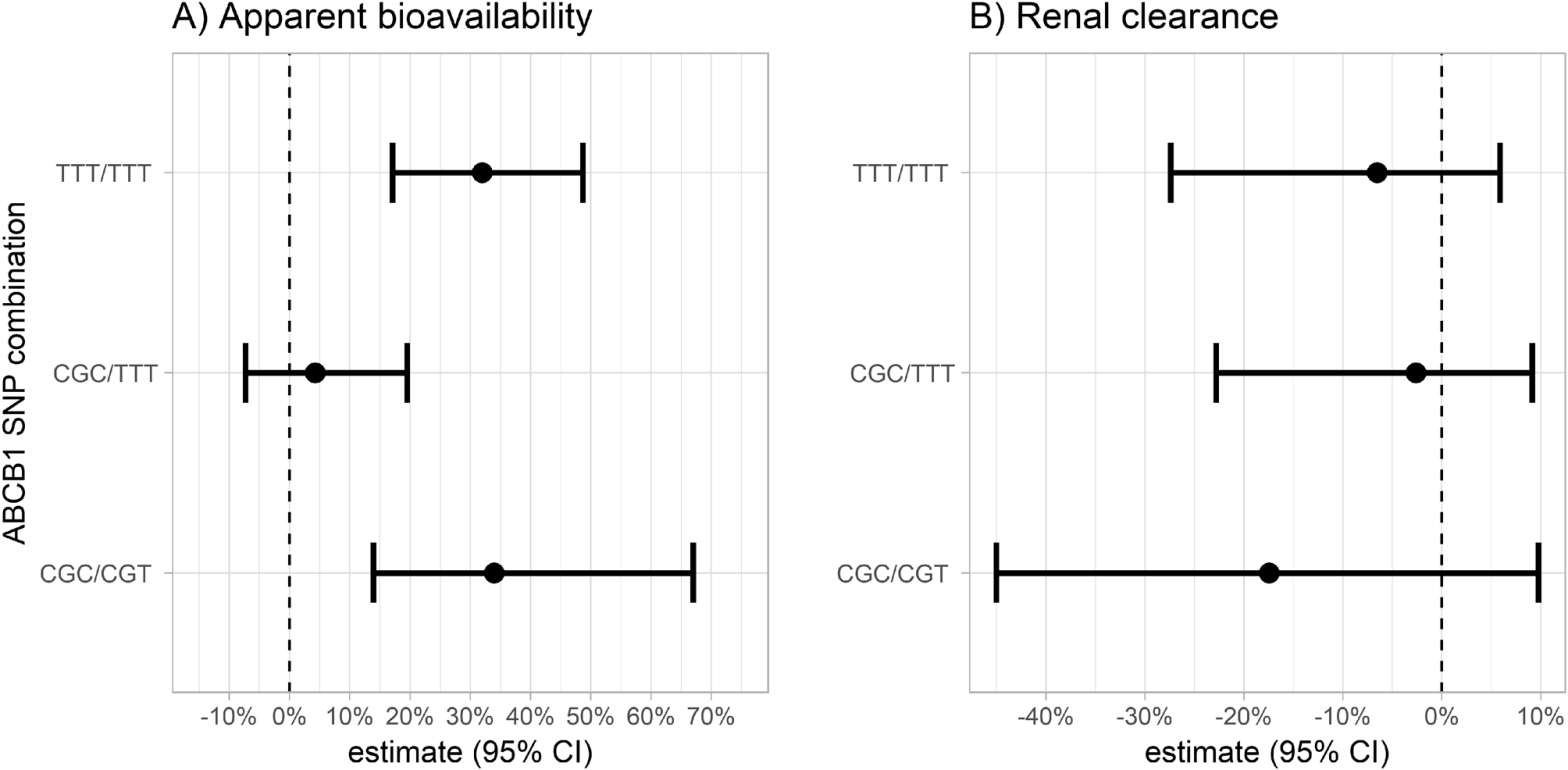
Relative difference in (**A**) apparent bioavailabilities and (**B**) renal clearance comparing different SNP combination groups to the reference SNP combination CGC/CGC. Median and 95% confidence intervals (95% CI) of fixed effects parameter estimates obtained from a bootstrap. The vertical dashed line represents no difference compared to the reference SNP combination. Refer to Table 3 for further information.

Apparent bioavailability was estimated separately for each of the defined SNP combinations in the population pharmacokinetic model, resulting in a significant drop in OFV by 26.1 points. In addition, renal clearances (CL_R_), zero-order absorption durations (D2) and Ka were also computed separately for each SNP combination. However, the model did not improve significantly (drop in OFV by 1.27, 3.44 and 3.55 points, respectively). A non-parametric bootstrap with 1,000 samples was conducted for the model with separately estimated apparent bioavailabilities in each SNP combination group. Relative differences in apparent bioavailabilities and renal clearance between SNP combinations are summarized in Fig. 1. CGC/CGT and TTT/TTT carriers had an approximately 35% higher bioavailability compared to CGC/CGC, while CGC/TTT carriers had a similar bioavailability compared to CGC/CGC. However, no significant differences in renal clearance were observed among different SNP combination groups.

**Figure 2:**
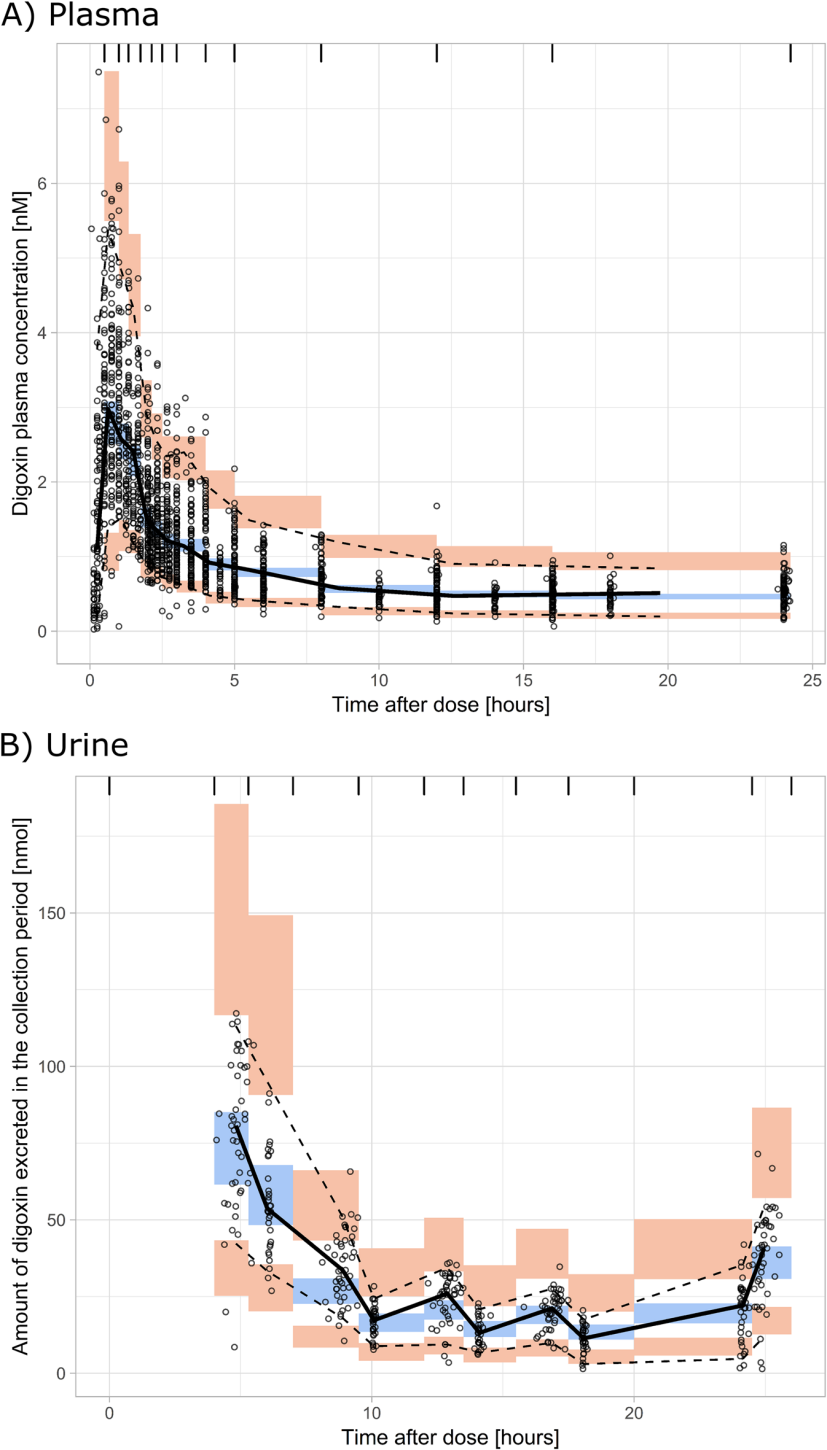
Visual predictive check of the final model (model 4) of (**A**) plasma, (**B**) urine. Solid (dashed) lines represent medians (5%, 95% percentiles) of observed concentrations; orange, blue and orange areas represent 95% confidence intervals of 5%, 50% and 95% percentiles predicted by the model. For a correctly specified compartmental model, observed medians should lie inside the middle blue boxes. Observed 95% percentiles should lie within the upper and 5% percentiles within the lower orange boxes.

**Figure 3:**
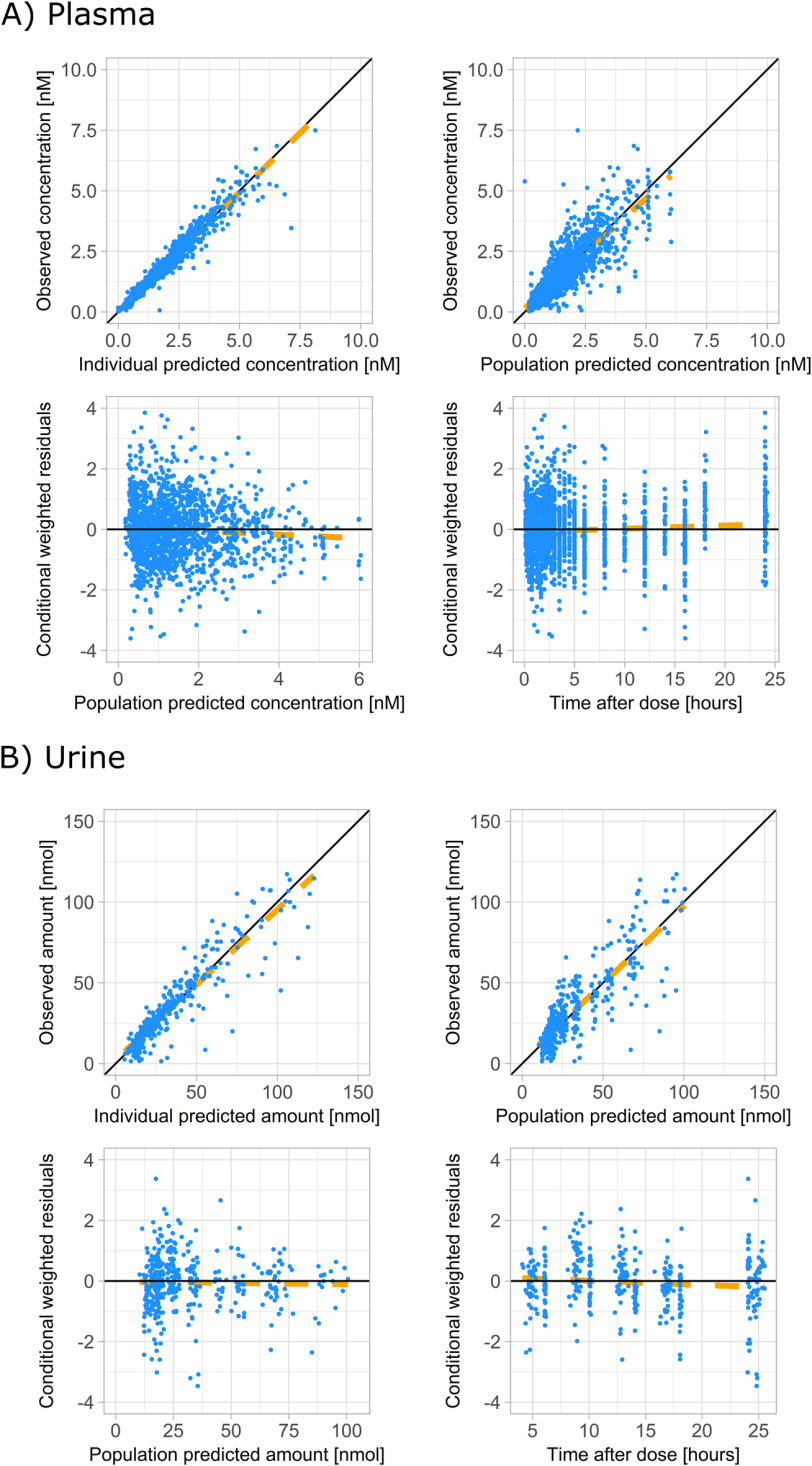
Goodness-of-fit plots of final model (model 4) of (**A**) plasma concentrations and (**B**) urine concentrations.

The final point estimates and bootstrap statistics of pharmacokinetic parameters are summarized in Table 3. VPC and Goodness of Fit (GOF) plots of plasma and urine data are shown in Figs. 2 and 3.

**Table 3:** Final model parameter estimates (model 4).

Pharmacokinetic parameter	Symbol	Point estimate (IIV CV%)	Bootstrap median (95% CI)
Non-renal clearance (L/h)	CL_NR_	0.254 (–)	0.251 (0.0232 to 0.257)
Renal clearance (L/h)	CL_R_	8.08 (32.8)	8.00 (7.17 to 8.91)
Apparent central volume of distribution (L)	V2/F	109 (15.7)	107 (92.1 to 121)
Intercompartmental clearance (L/h)	Q/F	61.1 (10.3)	60.1 (54.1 to 67.8)
Apparent peripheral volume of distribution (L)	V3/F	774 (17.9)	765 (682 to 879)
Zero-order absorption duration (h)	D2	0.536 (27.8)	0.521 (0.464 to 0.577)
Lag time for zero order absorption (h)	ALAG2	0.0955 (48.0)	0.0940 (0.0781 to 0.105)
First order absorption rate constant (1/h)	Ka	0.636 (68.5)	0.658 (0.500 to 1.04)
Lag time for first order absorption (h)	ALAG1	2.43 (11.8)	2.42 (2.20 to 2.71)
Percentage of first order absorption	FF1	0.267 (18.4)	0.278 (0.217 to 0.343)
Relative difference in bioavailability in trial I compared to trial II	BIO	− 0.303 (13.1)	− 0.304 (− 0.392 to − 0.218)
Relative difference in bioavailability for CGC/CGT compared to the CGC/CGC ABCB1 SNP combination group	BIOH2	0.350 (–)	0.339 (0.131 to 0.672)
Relative difference in bioavailability for CGC/TTT compared to the CGC/CGC ABCB1 SNP combination group	BIOH3	0.0613 (–)	0.0421 (− 0.0757 to 0.203)
Relative difference in bioavailability for TTT/TTT compared to the CGC/CGC ABCB1 SNP combination group	BIOH4	0.348 (–)	0.320 (0.167 to 0.490)
First order absorption rate constant of test period in trial II (1/h)	KAT	0.201 (–)	0.197 (0.155 to 0.263)

IIV CV%, coefficient of variation on inter-individual variability; 95%CI, 95% confidence interval.

## Discussion

In this evaluation based on a detailed characterization of digoxin pharmacokinetics in healthy volunteers, *ABCB1* SNP combinations had a significant albeit small influence on the (apparent) bioavailability but not on the renal elimination of the drug. This result is in line with a previous non-compartmental analysis (NCA) of one of the studies included in this population pharmacokinetic evaluation, where C_max_ and AUC_0–24 h_ of digoxin were significantly higher in CGC/CGT and TTT/TTT, but not in CGC/TTT carriers, compared to CGC/CGC^52^. In contrast, in a study by Xu et al. subjects carrying TTT/TTT showed a higher average C_max_ and AUC_0–4h_^31^ compared to CGC/CGC in a Chinese Han population, but the observed difference was not statistically significant. In previous studies, no systematic evaluation of the effect of CGT on the pharmacokinetics of digoxin has been conducted. Several previous studies showed the effect of 3435CT on the pharmacokinetics of digoxin. However, we cannot identify the subjects as belonging to the CGT group since the SNPs at positions 1236 and 2677 were not considered. Therefore, there is currently not enough data to support a genotype effect of CGT. Kim et al. included CGC/CGT carriers in an *ABCB1* genotype evaluation regarding the pharmacokinetics of fexofenadine. However, no statistical evaluation was conducted since there was only a single CGC/CGT carrier. We cannot explain why in our evaluation homozygous (but not heterozygous) carriers of TTT as well as the CGC/CGT genotype group had a different apparent bioavailability compared to homozygous wildtype carriers. Beyond the relatively small effects of the combined genetic variants and the pronounced inter-individual variability of apparent bioavailability, other possible explanations would include (1) the mechanisms by which each single SNP and/or the SNP combination may modify overall P-gp expression/activity; indeed, an effect of C3435T could be attenuated by the presence of the two further SNPs studied here; (2) whether a dominant model, a co-dominant model or a recessive model would be most appropriate to describe the relationship between genotype and P-gp expression/activity; for the presence of all three SNPs (TTT group), a recessive model would be in agreement with our findings; (3) effects of other covariates not assessed in the present analysis, such as further SNPs of ABCB1, SNPs in xenobiotic receptors involved in P-gp regulation etc.; (4) a chance finding based on the relatively low number of subjects in the CGC/CGT group (n = 5); this needs further investigation, and to this end, it would be interesting to assess individuals with two respective mutated alleles (CGT/CGT) but these were not present in our population. Although the employed model is of an empirical nature, net apparent bioavailability reflects the sum of intestinal absorption and intestinal as well as biliary secretion, which presumably results in enterohepatic circulation. The 35% higher bioavailability in TTT/TTT and in CGC/CGT carriers thus cannot be translated directly into a 35% lower activity of P-gp in these individuals. The results also do not allow to draw further conclusions on the underlying molecular mechanisms, such as reduction in ATP binding affinity, loss of ATP hydrolysis, modification of protein folding, or reduction in P-gp expression^46^. Published data on the effect of the TTT SNP combination on P-gp expression in the duodenum or the intestine is equivocal^53^. Although there are contradictory data on whether ABCB1 genotype would influence P-gp activity in vitro or in vivo, the present study showed an effect of combined SNPs on digoxin apparent bioavailability along with the absence of a respective effect on renal clearance of digoxin. This casts further doubts on the role of P-gp as a rate-limiting transporter for digoxin elimination in the kidney. However, we cannot rule out that our limited sample size was insufficient to detect minor differences in renal clearance between groups. Also, in our previous NCA analysis, no significant difference in renal clearance was found between CGC/CGC and other SNP combination groups^52^. When digoxin was given intravenously or orally with a strong P-gp inducer, renal elimination of digoxin was not relevantly affected, but the AUC_0–144 h_ for both administration routes and AUC_0–3 h_, C_max_, and bioavailability for oral administration were decreased. In addition, non-renal clearance (which might reflect intestinal and/or biliary clearance) for intravenous administration and t_max_ for oral administration were increased^19^. These findings also indicate that P-gp activity is not rate-limiting for the elimination of digoxin in the kidney. However, the impact of SLCO4C1 polymorphisms on the pharmacokinetics of substrates is unknown^54^. Whether SLCO4C1 (cytogenetic location: 5q21.1) is in linkage disequilibrium with ABCB1 (cytogenetic location: 7q21.12) has not been investigated yet. Therefore, we assume that polymorphisms of SLCO4C1 are randomly distributed in our study subjects, while we cannot exclude whether such polymorphisms may have an additional impact on the pharmacokinetics of digoxin.

In our empirical pharmacokinetic model, different values for apparent bioavailability were estimated for the two trials due to differences in study designs (see Table 3), probably attributable to the use of different concomitantly administered drugs. Thus, correcting for trial-specific differences was necessary to allow identification of trial-independent effects of *ABCB1* SNP combinations. Furthermore, we introduced a mixed zero- and first-order absorption model since some plasma concentration profiles exhibited a double peak phenomenon, with a second peak occurring after 4 h to 8 h post-dose. The double peak phenomenon could not be attributed to a food effect, but is in line with previous evaluations of digoxin exhibiting a similar phenomenon^55,56^.

Another limitation of this study is that plasma and urine samples were only available up to 24 h after drug administration, which covers less than one elimination half-life of digoxin. However, the assessment of drug elimination should be reliable once absorption is completed. Indeed, the estimated renal clearance of digoxin was comparable with previously published data^57^. Despite the limitations, the chosen population pharmacokinetic approach allowed to correct for differences between trials and to describe the effect particularly attributable to SNP combinations based on a large and detailed dataset.

In the previous studies assessing the effect of *ABCB1* genotypes on digoxin pharmacokinetics, noncompartmental methods were used, including C_max_, AUC_0–t,_ time of maximum plasma concentration (t_max_) or CL_R_^27–35^. In general, a population pharmacokinetic evaluation might be advantageous to identify effects on parameters more closely related to physiological processes. Although our model described the data well, the complex interplay of drug absorption, intestinal secretion and biliary elimination could not be captured in detail by the empirical model solely based on oral administration of digoxin. The information on genotype effects that could be obtained by the present evaluation turned out to be supportive but not superior to the information obtained by noncompartmental analysis. Using semiphysiological models applied to datasets including both oral and intravenous administration, as has been used for drugs undergoing first pass metabolism such as midazolam^58^, might be a promising approach to learn more about the mechanism of *ABCB1* genotype effects on digoxin pharmacokinetics.

Whether the observed relationship between the SNP combinations/haplotypes of ABCB1 and digoxin can be transferred to other P-gp substrates is currently not clear. For instance, in a previous study conducted in a Chinese population, 1236CC carriers had a lower C_max_ (− 53%; *p* = 0.013), AUC_0-∞_ (− 40%; *p* = 0.04), and cumulative amount excreted in urine over 6 h (− 52%; *p* = 0.027) and a higher apparent oral clearance (+ 35%; *p* = 0.013) of cloxacillin, another P-gp substrate, compared to carriers of 1236CC and 1236CT. Moreover, the homozygous CGC carriers also had a lower C_max_ (*p* = 0.017), AUC_0–∞_ (*p* = 0.032), and cumulative amount excreted over 6 h in urine (*p* = 0.026) and a higher apparent oral clearance of cloxacillin (*p* = 0.002) compared to homozygous carriers of TTT. Renal clearance of cloxacillin was not altered by the SNP combination TTT/TTT^59^. This result is very similar to the one obtained for digoxin. In contrast, in a study in Japanese subjects, homozygous carriers of TTT (defined as homozygous ABCB1*2 in the corresponding publication) compared to subjects who did not carry TTT had a lower renal clearance (*p* < 0.05) und urinary recovery (*p* < 0.01) of the P-gp substrate irinotecan and its metabolites, while there was no significant difference in the ratio AUC_0–∞_/dose^60^.

Furthermore, Kim et al. showed that homozygosity for TTT (defined as homozygous ABCB1*2 in the corresponding publication) was related to a difference in AUC_0–16 h_ of orally administrated fexofenadine, another P-gp probe substrate, in the opposite direction: The AUC was 40% lower compared to homozygous CGC (defined as homozygous ABCB1*1/*1)^47^, suggesting increased intestinal secretion for this SNP combination. Moreover, the effect of different *ABCB1* SNP combinations on the pharmacokinetics of cyclosporine was also inconsistent in previous reports, which may be attributable to the high variability in the pharmacokinetics in the heart and renal transplantation patients^61–64^. As a potential explanation for discrepant findings, digoxin was suggested to bind to different sites of P-gp unlike other typical P-gp substrates^65^. Whether the SNP combination of P-gp alters the structure of the binding site for digoxin, but not for other substrates, is also unknown. Thus, the whole protein structure of different P-gp SNP combinations and related drug-protein binding needs further to be studied in the future.

Additionally, the DNA methylation level in the *ABCB1* promoter may also influence the *ABCB1* activity. Wu et al. evaluated both the effect of the SNP combination of *ABCB1* and DNA methylation level in the *ABCB1* promoter on digoxin pharmacokinetics. mRNA expression in intestinal epithelial cells showed no difference between homozygous CGC and homozygous TTT carriers (*p* = 0.087). However, mRNA expression of homozygous TTT-HM carriers, who had a higher degree of DNA methylation, was significantly decreased compared to homozygous TTT-LM carriers (lower degree of DNA methylation), homozygous CGC-LM and homozygous CGC-HM carriers by 31.1, 27.9 and 43.6% (*p* = 0.02, 0.013 and 0.008), respectively. Subjects who carried homozygous TTT–HM had a significant higher AUC_0–72 h_, AUC_0–∞,_ C_max_ and lower apparent oral clearance compared to homozygous CGC-LM (*p* < 0.05)^40^. These results suggest that DNA methylation should be considered as a covariate when assessing the effect of different *ABCB1* SNP combinations on the pharmacokinetics of digoxin.

A further method to analyze the activity of membrane transporters is to evaluate the concentration of endogenous substrates (e.g., coproporphyrin I and III for OATP1B1/1B3 transporter)^66,67^. Recently, testosterone, aldosterone, and cortisol have been demonstrated to be endogenous substrates of P-glycoprotein in vitro^7,68^. In vivo, the genotype of P-gp affected the elimination of aldosterone in urine, which might be a potential biomarker for evaluating renal P-gp activity^68^. Thus, endogenous markers might help to further characterize the functional role of *ABCB1* SNP combinations.

## Conclusion

The empirical population pharmacokinetic evaluation showed that homozygous carriers of the TTT and CGC/CGT have a 35% higher apparent bioavailability of oral digoxin, while no effects of CGC/TTT on apparent bioavailability and of any *ABCB1* variants on renal elimination were observed. These results support the use of digoxin as a phenotyping substrate of intestinal but not of renal P-gp activity. Our study suggests considering the effect of a combination of SNPs on the pharmacokinetics of digoxin, rather than focusing on single SNPs. This might play an important role in the design and refinement of transporter phenotyping studies, including the development of appropriate sampling schedules, and the mode of administration. Furthermore, protein expression, protein structure and/or P-gp affinity to digoxin or other substrates in different genotypes need to be further investigated.

## Methods

### Clinical trials

Data from 40 healthy Caucasian subjects receiving single oral doses of 0.5 mg digoxin in two clinical trials were analyzed^52,58^. In trial 1, a single dose of 0.5 mg digoxin was given concomitantly with 2 mg oral midazolam, 1 mg intravenous midazolam, 125 mg tolbutamide, 150 mg caffeine, 20 mg omeprazole and 30 mg dextromethorphan in the reference period. In the test period, the drugs of the reference period were combined with ethanol^58^. In trial 2, a single dose of 0.5 mg digoxin was given alone in the reference period and in combination with 10 mg adefovir, 500 mg metformin, 2 mg pitavastatin, and 100 mg sitagliptin in the test period^52^. Blood and urine samples were collected up to 24 h after drug administration. The study design and the timing of blood and urine samples are summarized in Table 4.

**Table 4:** Summary of study designs used for pharmacokinetic analysis of digoxin.

Study	Study design	Number of subjects	Pharmacokinetic sampling	Dose regimen of digoxin	Identity and manufacturer of digoxin
Trial I^58^	Reference period: **digoxin** 0.5 mg + midazolam 2 mg p.o. + midazolam 1 mg i.v. + tolbutamide 125 mg + caffeine 150 mg + omeprazole 20 mg + dextromethorphan 30 mg Test period: as above + ethanol (up to 0.7 ‰ C_max_)	16 healthy Caucasian subjects (male = 8; female = 8)	Plasma (28 samples): -0:15 h predose, and postdose at 0:08, 0:20, 0:30, 0:45, 1:00, 1:15, 1:30, 1:45, 2:00, 2:05, 2:08, 2:20, 2:30, 2:45, 3:00, 3:30, 4:00, 4:30, 5:00, 6:00, 8:00, 10:00, 12:00, 14:00, 16:00, 18:00 and 24:00 h Urine (6 collection interval): Predose, 0–6 h 6–10 h, 10–14 h, 14-18 h and 18-24 h	Single dose (2 tablets)	Digacin 0.25 mg Mibe GmbH Arzneimittel, Brehna, Germany
Trial II^52^	Reference period: **digoxin** 0.5 mg Test period: **digoxin** 0.5 mg + adefovir 10 mg + metformin 500 mg + pitavastatin 2 mg + sitagliptin 100 mg	24 healthy Caucasian subjects (Male = 10; Female = 14)	Plasma (19 samples): -0:15 h predose, and postdose at 0:15, 0:30, 0:45, 1:00, 1:20, 1:40, 2:00, 2:20, 2:40, 3:00, 3:30, 4:00, 5:00, 6:00, 8:00, 12:00, 16:00 and 24:00 h Urine (6 collection interval): Predose, 0–4 h, 4–8 h, 8–12 h, 12–16 h and 16–24 h	Single dose (2 tablets)	Digacin 0.25 mg Mibe GmbH Arzneimittel, Brehna, Germany

C_max,_ maximal observed plasma concentration.

Genotyping of *ABCB1* was carried out using the DMET Plus Array (Affymetrix, Santa Clara, California, United States)^69^. To define SNP combination groups, the common *ABCB1* SNPs C1236T, G2677T and C3435T were taken into account^49^. Deviation from Hardy–Weinberg equilibrium was assessed using a chi square test.

Blood and urine samples of digoxin were processed with solid-phase extraction (Strata-X 30 mg/3 mL, product number: 8B-S100-TBJ, Phenomenex, Aschaffenburg, Germany). Digoxin-d3 was spiked in blood and urine sample as internal standard (product code: TRC-D446577-2.5MG, Toronto Research Chemicals, Toronto, Canada). Concentrations were quantified with a validated high-performance liquid chromatography-tandem mass spectrometry method (Agilent 1,260 Infinity, Agilent Technologies, Waldbronn, Germany/API 5,000, AB Sciex Germany GmbH, Darmstadt, Germany) as described previously^52,58^. The calibration range of digoxin in plasma and urine was 0.128 nmol/L to 38.4 nmol/L and 1.28 nmol/L to 384 nmol/L, respectively. All assays fulfilled the bioanalytical method validation criteria according to the FDA and the EMA guidelines^70,71^. Intra-day and inter-day inaccuracy and imprecision of all quality control samples were < 15%.

### Data analysis

#### Digoxin empirical model

2083 plasma and urine samples were included for the population pharmacokinetics analysis. All samples with concentrations below LLOQ were pre-dose samples and were therefore omitted from the pharmacokinetic (PK) analysis (2.1% of all observations). In one subject, no digoxin was detectable in plasma and urine samples in the reference period in trial 2, suggesting non-compliance despite a mouth check for oral administration. Therefore, samples for this subject and period were not included in the analysis (1.1% of total observations).

A population pharmacokinetic model was developed using NONMEM 7.4.2 (ICON Development Solutions, Ellicott City, MD, USA) with the first-order conditional estimation method with interaction option (FOCE + I). Data visualization was performed using R 3.4.2 (R Foundation for Statistical Computing, Vienna, Austria) and the tidyverse R package^72^. Model diagnostics were carried out using XPOSE 4.5.0.17^73^.

The empirical model was developed by starting with a one-compartment model with linear elimination and increasing the model complexity step-wise. Different absorption models were tested, including zero-order, first-order with/ without lag time and enterohepatic cycling (EHC), to describe observed double peak phenomena. Clearance and volume of distribution parameters were scaled allometrically with body weight. Inter-individual variability (IIV) and between occasion variability (BOV) were computed for all pharmacokinetic parameters. A combined proportional and additive error model was applied. To account for potential systematic differences between trials, study effects were tested on all fixed effects parameters.

For all modelling steps, changes in OFV, the Akaike information criterion (AIC) for non-nested models, GOF and VPC were considered. For statistical tests based on the OFV, a chi squared distribution with the appropriate number of degrees of freedom was assumed. Furthermore, a non-parametric bootstrap analysis with 1,000 samples was conducted.

#### Effect of different *ABCB1* SNP combinations

After identification of a reasonable base model, relationships between SNP combinations and pharmacokinetic parameters were evaluated by introducing different Ka, D2, apparent bioavailabilities and CL_R_ in each SNP combination group in the population pharmacokinetic model. The changes in OFV were considered to identify significant relationships.

### Compliance with ethical standards

Both clinical trials were approved by the Ethics Committee of the Faculty of Medicine, University of Cologne, Germany, and conducted in accordance with applicable regulations and the ethical principles described in the Declaration of Helsinki and the International Conference on Harmonization guidelines for Good Clinical Practice. The clinical trial I and clinical trial II are registered at clinicaltrials.gov with the IDs NCT02515526 and NCT02743260, respectively. Informed consent was obtained from all participants.

## Supplementary information

Supplementary information

## Data Availability

The datasets analyzed during the current study are available from the corresponding author on reasonable request.
