# Supplementary material for: Combinations of common SNPs of the transporter gene ABCB1 influence apparent bioavailability, but not renal elimination of oral digoxin
Source: Sci Rep. 2020 Jul 27;10:12457. doi: 10.1038/s41598-020-69326-y (PMC7385621; doi:10.1038/s41598-020-69326-y)
Supplement: Supplementary file 1 — Supplementary information [file 41598_2020_69326_MOESM1_ESM.pdf]

TITLE PAGE OF SUPPLEMENTARY INFORMATION

Title: Combinations of Common SNPs of the transporter gene ABCB1 influence apparent bioavailability, but not renal elimination of oral digoxin

Author: Chih-hsuan Hsin (1), Marc S. Stoffel (1), Malaz Gazzaz (1, 4), Elke Schaeffeler (2,3), Matthias Schwab (2,5), Uwe Fuhr (1), Max Taubert* (1)

Institution:

(1) University of Cologne, Faculty of Medicine and University Hospital Cologne, Department I of Pharmacology, Cologne, Germany

(2) Dr. Margarete-Fischer-Bosch Institute of Clinical Pharmacology, Stuttgart, Germany

(3) University of Tuebingen, Tuebingen, Germany

(4) Department of Clinical Pharmacy, College of Pharmacy, Umm Al-Qura University, Makkah, Saudi Arabia

(5) Department of Clinical Pharmacology and Department of Pharmacy and Biochemistry, University of Tuebingen, Tuebingen, Germany University Hospital Tuebingen, Germany

Corresponding author contact information:

Dr. med. Max Taubert

Department I of Pharmacology, Center for Pharmacology, Clinical Pharmacology Unit

University Hospital Cologne (AöR)

Gleueler Strasse 24, 50931 Köln, Germany

E-mail: max.taubert@uk-koeln.de

Phone: +49-(0)-221-478-86716

Fax: +49-(0)-221-478-7011

Table S1. Effect of *ABCB1* Haplotype or tagging SNPs on digoxin PK (as reported in literature)

Dosage	Number of subjects	Parameter	Effect	Ref.
**Single SNP**				
**c.1236 C>T**				
0.25mg<br>single dose	Chinese Han<br>20 healthy subjects	C_{max} (µg/L)	CC (1.32; 1.04 - 3.10) = CT (1.16; 0.80 - 2.33)<br>= TT (1.83; 1.35 - 2.33)	[31]
		AUC_{0-4h} (ng*h/mL)	CC (2.98; 2.46 - 3.42) = CT (2.49; 1.73 - 3.13)<br>= TT (3.11; 2.71 - 3.57)	
		t_{max} (h)	CC (1.0; 0.66 - 2.0) = CT (1.0; 0.66 - 1.5)<br>= TT (0.66; 0.66 - 1.0)	
0.5mg<br>single dose	Tunisian population<br>58 atrial fibrillation patients	C_{6h} (ng/mL)	CC (0.97 ± 0.43) = CT (1.13 ± 0.46)= TT (0.94 ± 0.16)	[33]
0.59± 0.33 mg<br>Mean daily dose	175 subjects[#]	Mean difference in serum concentration (ng/mL)	**CC (Ref) < CT* (+0.28, 0. 07 - 0.48)**<br>**CC (Ref) < TT* (+0.39, 0.14 - 0.64)**	[35]
0.5mg<br>single dose	16 healthy subjects[##]	AUC_{last}(ng*h/mL)	CC (7.33 ± 0.93) = CT (6.63 ± 2.50) = TT (9.59 ± 1.92)	[43]
**c.2677 G>T/A**				
0.25mg<br>single dose	Chinese Han<br>20 healthy subjects	C_{max} (ng/mL)	GG (1.25; 0.80 - 3.10) = GT/GA (1.25; 1.13 - 1.58)<br>= TT (1.83; 1.35 - 2.33)	[31]
		AUC_{0-4h} (ng*h/mL)	GG (2.48; 1.73 - 3.42) = GT/GA (2.64; 2.49 - 3.13)<br>= TT (3.11; 2-71 - 3.57)	
		t_{max} (h)	GG (1.0; 0.66 - 2.0) = GT/GA (0.83; 0.66 - 1.5)<br>= TT (0.66; 0.66 - 1.0)	
0.5mg<br>single dose	Tunisian population<br>58 atrial fibrillation patients	C_{6h} (ng/mL)	GG (1.02 ± 0.45) = GT (1.10 ± 0.46)<br>= TT (0.95 ± 0.06) = AA (1.06)[a]	[33]

0.59± 0.33 mg<br>Mean defined daily dose	175 subjects[#]	Mean difference in serum concentration (ng/mL)	**GG (Ref) < GT* (+0.29, 0.09 - 0.50)**<br>**GG (Ref) < TT* (+0.35, 0.09 - 0.61)**	[35]
0.25 mg daliy<br>steady state	Caucasian<br>15 congestive heart failure patients	$C_{min,ss}$ (ng/mL)	GG (0.555 ± 0.244) = GT/GA (0.550 ± 0.193)<br>= TT/TA (0.436 ± 0.358)	[30]
0.5 mg<br>single dose	Caucasians (n=25)<br>Africans (n=6)<br>Asian (n=1)<br>32 healthy subjects in total	C_{max} (ng/mL)<br>AUC_{0-4h} (ng*h/mL)<br>AUC_{0-24h} (ng*h/mL)<br>AUC_{0-48h} (ng*h/mL)<br>Ae_{0-48h} (µg)	GG (2.45 ± 0.61) = GT (2.64 ±0.74) = TT (2.95 ±0.84)<br>GG (5.8 ± 0.8) = GT (5.6 ± 1.4) = TT (6.8 ± 1.7)<br>GG (15.3 ± 2.2) = GT (15.4 ± 2.8) = TT (17.9 ± 4.6)<br>GG (22.2 ± 3.9) = GT (22.2 ± 3.8) = TT (26.6 ±6.8)<br>GG (151 ± 19) = GT (160 ± 26) = TT (171 ± 30)	[29]
1mg<br>single dose	"White" male<br>50 healthy subjects	C_{max} (ng/mL)<br>AUC_{0-4h} (ng*h /mL)<br>t_{max} (h)	GG (4.22) = GT/GA (3.91/4.00) = TT/TA (3.69/4.39)[a]<br>GG (9.06) = GT/GA (9.10/9.91) = TT/TA (8.95/10.5)[a]<br>GG (1.00) = GT/GA (1.00/0.85) = TT/TA (1.10/0.96)[a]	[27]
0.5mg<br>Single dose	Danish population<br>30 healthy subjects	AUC_{0-24h} (nmol*min /L)	GG (403; 336 - 470) = GT (409; 367 - 451)<br>= TT (403; 348 - 458)	[37]
0.5mg<br>single dose	16 healthy subjects[##]	AUC_{last} (ng*h/mL)	GG (7.54 ± 1.78) = GT (9.27 ± 2.81) = TA (8.03 ± 2.40)	[43]
**c.3435 C>T**				
0.25mg<br>single dose	Chinese Han<br>20 healthy subjects	C_{max} (ng/mL)<br><br>AUC_{0-4h} (ng*h/mL)<br><br>t_{max} (h)	CC (1.25; 0.80 - 3.10) = CT (1.25; 1.13 - 1.58)<br>= TT (1.83; 1.35 – 2.33)<br>CC (2.48; 1.73 - 3.42) = CT (2.64; 2.49 - 3.13)<br>= TT (3.11; 2.71 - 3.57)<br>CC (1.0; 0.66-2.0) = CT (0.83; 0.66 - 1.5)<br>= TT (0.66; 0.66 - 1.0)	[31]
0.5mg<br>single dose	Tunisian population<br>58 atrial fibrillation patients	C_{6h} (ng/mL)	CC (0.95 ± 0.37) = CT (1.13 ± 0.43)<br>= TT (1.12 ± 0.51)	[33]

0.59± 0.33 mg<br>Mean defined daily dose	175 subjects[#]	Mean difference in serum concentration (ng/mL)	**CC (Ref) < CT* (+0.27, 0.04 - 0.50)**<br>**CC (Ref) < TT* (+0.27, 0.02 - 0.52)**	[35]
0.25 mg daily<br>Steady state	Caucasian<br>15 congestive heart failure patients	$C_{min,ss}$ (ng/mL)	CC (0.655 ± 0.107) = CT (0.405 ± 0.185)<br>= TT (0.703 ± 0.518)	[32]
0.5 mg<br>single dose	Caucasians (n=25)<br>Africans (n=6)<br>Asian (n=1)<br>32 healthy subjects in total	C_{max} (ng/mL)<br>AUC_{0-4h} (ng*h/mL)<br><br>AUC_{0-24h} (ng*h/mL)<br><br>AUC_{0-48h} (ng*h/mL)<br><br>Ae_{0-48h} (µg)	CC (2.44 ± 0.59) = CT (2.42 ± 0.79) = TT (2.96 ±0.76)<br>**CC (5.7 ± 0.9) < TT* (6.7 ± 1.5);**<br>**CT (5.2 ± 1.4) < TT* (6.7 ± 1.5)**<br>**CC (14.9 ± 2.5) < TT* (17.9 ± 3.9);**<br>**CT (13.9 ± 2.6) < TT* (17.9 ± 3.9)**<br>**CC (21.8 ± 4.1) < TT* (26.2 ± 5.8) ;**<br>**CT(20.1 ± 3.6) < TT* (26.2 ± 5.8)**<br>CC (149 ± 18) = CT (158 ± 30) = TT (164 ± 30)	[29]
1mg<br>single dose	White male<br>50 healthy subjects	C_{max} (ng/mL)<br>AUC_{0-4h} (ng*h/mL)<br>t_{max} (h)	CC (4.12) = CT (3.87) = TT (4.14) [a]<br>CC (9.72) = CT (8.89) = TT (9.51) [a]<br>CC (0.92) = CT (1.05) = TT (0.95) [a]	[27]
0.25 mg<br>single dose	Japanese<br>15 healthy subjects	C_{1h} (ng/mL)<br>C_{4h} (ng/mL)<br>C_{12h} (ng/mL)<br>AUC_{0-4h} (ng*h/mL)<br><br>AUC_{0-24h} (ng*h/mL)	CC (2.00 ± 0.40) = CT (1.56 ± 0.47) = TT (1.49 ±0.36)<br>CC (0.58 ± 0.09) = CT (0.50 ± 0.10) = TT (0.37 ± 0.20)<br>CC (0.28 ± 0.19) = CT (0.15 ±0.17) = TT (0.11 ± 0.16)<br>**CC (4.11 ± 0.57) > CT* (3.20 ± 0.49);**<br>**CC (4.11 ± 0.57) > TT* (3.27 ± 0.58)**<br>CC (8.84 ± 3.28) = CT (5.80 ±1.94) = TT (5.74 ± 3.04)	[34]
0.25mg BID (Day 1-2),<br>0.25mg QD (Day 3-5)<br>Steady-state	Caucasians<br>24 healthy subjects	C_{max} (ng/mL)<br>AUC_{0-4h} (ng*h/mL)<br>Ae_{0-24h} (µg)<br>C_{trough} (ng/mL)	**CC (1.7 ± 0.2) < TT* (2.1 ± 0.4)**<br>**CC (4.6 ±0.7) < TT* (5.6 ± 0.9)**<br>**CC (79.1 ± 15.5) < TT* (94.7 ± 13.2)**<br>**CC (0.44 ± 0.07) < TT* (0.60 ± 0.16)**	[28]

		t_{max} (h)<br>AUC_{0-24h} (ng*h/mL)	**CC (1.0, 0.5 - 1.5) > TT* (0.8, 0.5 - 1.5)**<br>CC (15.5 ± 2.5) = TT (18.3 ± 4.1)	
0.25mg BID (Day 1-2),<br>0.25mg QD (Day 3-15)<br>Steady state	14 healthy subjects[##]	C_{max} (ng/mL)	**CC (-38%) < TT**, b**	[30]
0.5mg<br>Single dose	12 healthy subjects[##]	AUC_{0-4h} (ng*h/mL)<br>AUC_{0-24h} (ng*h/mL)<br>AUC_{0-48h} (ng*h/mL)<br>t_{max} (h)<br>C_{max} (ng/mL)<br>Ae_{0-24h} (mg)<br>CL_r (ng/mL)	**CC (6.10 ± 0.83) < TT (7.53 ± 1.23)**<br>**CC (16.8 ± 1.73) < TT (20.2 ± 2.14)**<br>CC (25.0 ± 3.33) = TT (29.3 ± 3.35)<br>CC (0.9; 0.5 - 2.0) = TT (0.9; 0.7 - 1.5)<br>CC (2.7 ± 0.8) = TT (3.3 ± 0.8)<br>CC (0.16 ± 0.02) = TT (0.16 ± 0.04)<br>CC (6.30 ± 0.74) = TT (5.51 ± 0.84)	[36]
0.5mg<br>Single dose	Danish population<br>30 healthy subjects	AUC_{0-24h} (nmol*min/L)	**CC (376; 364 - 387) < CT* (394; 353 - 433)**<br>**CC (376; 364 - 387) < TT* (418; 370 - 466)**	[37]
0.5mg<br>single dose	12 healthy subjects[##]	C_{max} (ng/mL)<br>AUC_{0-24h} (ng*h/mL)	CC (2.3 ± 0.4) = CT (2.2 ± 0.4) = TT (2.2 ± 0.5)<br>CC (15.1 ± 3.60) = CT (13.0 ± 2.55) = TT (13.9 ± 1.95)	[42]
0.5mg<br>single dose	16 healthy subjects[##]	AUC_{last} (ng*h/mL)	CC (7.18 ± 1.88) = CT (9.00 ± 2.63)	[43]
**Haplotype**				
**c.2677G>T/A+c.3435C>T**				
0.25mg<br>single dose	Chinese Han<br>20 healthy subjects	C_{max} (ng/mL)<br>AUC_{0-4h} (ng*h/mL)<br>t_{max} (h)	GC/GC (1.25; 0.80 - 3.10) = GC/TT (1.25; 1.13 -1.58) = TT/TT (1.83; 1.35 - 2.33)<br>GC/GC (2.48; 1.73 -3.42) = GC/TT (2.64; 2.49 - 3.13) = TT/TT (3.11; 2.71 - 3.57)<br>GC/GC (1.0; 0.66 - 2.0) = GC/TT (0.83; 0.66 - 1.5) = TT/TT (0.66; 0.66 - 1.0)	[31]

1mg<br>single dose	White male<br>50 healthy subjects	C_{max} (ng/mL)	GC/GC (3.84) = TT/TT (3.69)	[27]
		$AUC_{0\text{-}4h}$ (ng*h/mL)	GC/GC (9.06) = TT/TT (9.06)	
		t_{max} (h)	GC/GC (0.90) = TT/TT (1.11)	
0.25mg<br>Single dose	Japanese population<br>15 healthy subjects	C_{1h} (ng/mL)	**GC/GC (1.98 ± 0.23) > TT/TT* (1.44 ± 0.23)**<br>**GC/GC (1.98 ± 0.23) > AC/TT* (1.39 ± 0.23)**	[38]
		$AUC_{0\text{-}4h}$ (ng*h/mL)	GC/GC (4.00 ± 0.30) = TT/TT (3.34 ± 0.29)<br>**GC/GC (4.00 ± 0.30) > AC/TT* (2.99 ± 0.17)**	
0.5mg<br>Single dose###	Japanese population<br>15 healthy subjects	AUC_{oral} (ng*h/mL)	**GC/GC (20.6 ± 1.5) < GC/TT* (29.6± 4.2)**<br>**GC/GC (20.6 ± 1.5) < TT/TT* (35.1 ± 11.9)**	[39]
		AUC_{iv} (ng*h/mL)	GC/GC (32.6 ± 2.7) = GC/TT (35.7 ± 2.0)<br>= TT/TT (40.7 ± 6.9)	
		CL (mL/min/kg)	GC/GC (3.8 ± 0.6) = GC/TT (3.7 ± 0.3)<br>= TT/TT (3.2 ± 0.2)	
		CL/F (mL/min/kg)	**GC/GC (6.1 ± 1.1) < GC/TT* (4.5 ± 0.4)**<br>**GC/GC (6.1 ± 1.1) < TT/TT* (3.9 ± 0.7)**	
		$CL_{r\ oral}$ (mL/min/kg)	GC/GC (3.1 ± 0.6) = GC/TT (2.5 ± 0.3)<br>**GC/GC (3.1 ± 0.6) > TT/TT* (1.9 ± 0.5)**	
		$CL_{r\ iv}$ (mL/min/kg)	GC/GC (2.8 ± 0.3) = GC/TT (2.1 ± 0.6)<br>**GC/GC (2.8 ± 0.3) > TT/TT* (1.9 ± 0.1)**	
		$CL_{cr\ iv}$ (mL/min/kg)	GC/GC (1.3 ± 0.3) = GC/TT (1.5 ± 0.3)<br>= TT/TT (1.4 ± 0.4)	
		$CL_{cr\ oral}$ (mL/min/kg)	GC/GC (1.4 ± 0.3) = GC/TT (1.5 ± 0.2)<br>= TT/TT (1.4 ± 0.4)	
		$CL_{sec\ iv}$ (mL/min/kg)	GC/GC (1.3 ± 0.2) = GC/TT (1.0 ± 0.1)<br>**GC/GC (1.3 ± 0.2) > TT/TT* (0.5 ± 0.2)**	
		$CL_{sec\ oral}$ (mL/min/kg)	**GC/GC (1.7 ± 0.3) > GC/TT (1.0 ± 0.3)**<br>**GC/GC (1.7 ± 0.3) > TT/TT* (0.7 ± 0.3)**	

		CL_{nr} (mL/min/kg)	GC/GC (1.2 ± 0.2) = GC/TT (1.2 ± 0.1) = TT/TT (1.4 ± 0.2)	
		F (%)	GC/GC (67.6 ± 4.3) = GC/TT (80.9 ± 8.9)<br>**GC/GC (67.6 ± 4.3) < TT/TT* (87.1 ± 8.4)**	
**c.1236C>T+c.2677G>T/A+c.3435C>T**				
0.25mg<br>single dose	Chinese Han<br>20 healthy subjects	C_{max} (ng/mL)	**TGC/CGC (1.04, 0.80 - 1.25) < TTT/TTT* (1.83, 1.35 - 2.33)**	[31]
		AUC_{0-4h} (ng*h/mL)	**TGC/CGC (2.14, 1.73 - 2.48) < TTT/TTT* (3.11, 2.71 - 3.57)**	
		t_{max} (h)	**TGC/CGC (1.0, 0.66 - 1.00) > TTT/TTT* (0.66, 0.66 - 1.00)**	
0.5mg<br>Single dose	Chinese Han<br>43 healthy subjects<br>(CGC/CGC-LM: CGC/CGC haplotype with a lower DNA methylation levels of ABCB1 promoter);<br>(TTT/TTT-HM: TTT/TTT haplotype with a higher DNA methylation levels of ABCB1 promoter)	AUC_{0-72h} (ng*h/mL)	**CGC/CGC-LM (21.3 ± 6.42) < TTT/TTT-HM (29.1 ± 7.13)**	[40]
		$AUC_{0-\infty}$ (ng*h/mL)	**CGC/CGC-LM (32.3 ± 7.26) < TTT/TTT-HM (41.7 ± 11.0)**	
		C_{max} (ng/mL)	**CGC/CGC-LM (1.98 ± 0.55) < TTT/TTT-HM (2.64 ± 0.67)**	
		t_{max} (h)	CGC/CGC-LM (0.97 ± 0.38) = TTT/TTT-HM (0.93 ± 0.25)	
		$t_{1/2}$ (h)	CGC/CGC-LM (36.4 ± 20.8) = TTT/TTT-HM (36.9 ± 19.7)	
		CL/F (L/h)	**CGC/CGC-LM (18.2 ± 4.74) > TTT/TTT-HM (15.0 ± 4.83)**	

Summary of *ABCB1* genotype/ haplotype effects on digoxin pharmacokinetic parameters from the literature. The genotype/ haplotype effects on the pharmacokinetic parameters are shown as mean ± standard deviation, mean with 95 % confidence interval or median with range (value of t_{max} and reference 31). [a] Only the mean has been reported. [b] Only the average difference between the two genotype groups has been reported. If the pharmacokinetic parameter showed statistically significant differences, the genotype/ haplotype effect is presented in **bold** in the table. *P<0.05; **P<0.01, [#] Subjects were recruited from the Rotterdam study, which was a prospective population-based cohort study in the city of Rotterdam, the Netherland. Details of the ethnicity on this population were not reported. [##] Details of the ethnicity on the population were not reported. [###] Digoxin was administrated orally and intravenously. C_{max},

maximal observed plasma concentration; $AUC_{0-t\ hour}$, area under the curve from 0−t hours; t_{max}, time of maximum plasma concentration; Ae_{0-24h}, amount excreted in urine within 24 hours; $C_{min,ss}$, minimum concentration at steady-state; C_{trough}, lowest concentration reached by a drug before the next dose is administered C_{1h}, C_{4h}, C_{6h}, C_{12h}, Serum concentration at 1, 4 , 6, 12 hours post-dose; CL, clearance clearance; CL_r, renal clearance; CLc_r, creatinine clearance; CL_{nr}, nonrenal clearance; CL_{sec}, apparent tublar secretory clearance; F, measured oral bioavailability
